# Effects of Ambient Bright Light on Neurobehavioral Symptoms in Dementia: A Systematic Review

**DOI:** 10.1093/geroni/igaa057.527

**Published:** 2020-12-16

**Authors:** Ying-Ling Jao, Julian Wang, Yo-Jen Liao, Diane Berish, Kimberly Van Haitsma, Marie Boltz

**Affiliations:** 1 Pennsylvania State University, University Park, Pennsylvania, United States; 2 Penn State University, University Park, Pennsylvania, United States

## Abstract

Most persons living with dementia (PLwD) experience behavioral and psychological symptoms of distress in dementia (BPSDs). Despite increased utilization of bright light to improve BPSDs, the evidence of effectiveness and dosage using ambient light has not been comprehensively examined. This review synthesized research evidence on the effect of ambient light on BPSD in PLwD. A literature search conducted in Scopus, PubMed, CINAHL, and Web of Science included keywords: dementia, bright light, ambient light, indirect light, behavior symptoms, agitation, wandering, depression, aggression, and apathy. Original studies that examined the effect of ambient light on BPSDs were included. Six studies were identified. Sample size ranged from 6 to 189. Lighting delivery methods included a lighting table and ceiling-mounted fixtures in public areas and/or participant’s bedroom. Lighting intensity ranged from 1,000-3,000 lux, color temperature ranged from bluish white to warm white, and exposure duration ranged from 4 to 24 hours a day. PLwD with higher light exposure showed more pleasure and alertness. Ambient bright lighting showed mixed results in reducing agitation with one study reporting increased agitation. Three out of four studies showed positive effects upon depressive symptoms. Ambient bright light positively impacted pleasure and alertness. Mixed results on agitation and depressive symptoms may be explained by differences in illuminance, color, duration, and targeted lighting positions. Further studies are needed to confirm the positive effects of ambient light on BPSD. Accurate lighting exposure measurements related to spectral compositions and dosage for individual PLwD would help explain the underlying relationships between lighting and BPSD.

